# P2X Receptors: Potential Therapeutic Targets for Symptoms Associated With Lung Cancer — A Mini Review

**DOI:** 10.3389/fonc.2021.691956

**Published:** 2021-06-29

**Authors:** Yonglin Mai, Zhihua Guo, Weiqiang Yin, Nanshan Zhong, Peter V. Dicpinigaitis, Ruchong Chen

**Affiliations:** ^1^ Department of Allergy and Clinical Immunology, State Key Laboratory of Respiratory Disease, National Clinical Research Center for Respiratory Disease, Guangzhou Institute of Respiratory Health, The First Affiliated Hospital of Guangzhou Medical University, Guangzhou, China; ^2^ Department of Thoracic Surgery, State Key Laboratory of Respiratory Disease, National Clinical Research Center for Respiratory Disease, Guangzhou Institute of Respiratory Health, The First Affiliated Hospital of Guangzhou Medical University, Guangzhou, China; ^3^ Department of Medicine, Albert Einstein College of Medicine & Montefiore Medical Center, Bronx, NY, United States

**Keywords:** ATP, P2X receptors, antagonist, cancer-related symptoms, tumor microenvironment

## Abstract

Symptoms associated with lung cancer mainly consist of cancer-associated pain, cough, fatigue, and dyspnea. However, underlying mechanisms of lung cancer symptom clusters remain unclear. There remains a paucity of effective treatment to ameliorate debilitating symptoms and improve the quality of life of lung cancer survivors. Recently, extracellular ATP and its receptors have attracted increasing attention among researchers in the field of oncology. Extracellular ATP in the tumor microenvironment is associated with tumor cell metabolism, proliferation, and metastasis by driving inflammation and neurotransmission *via* P2 purinergic signaling. Accordingly, ATP gated P2X receptors expressed on tumor cells, immune cells, and neurons play a vital role in modulating tumor development, invasion, progression, and related symptoms. P2 purinergic signaling is involved in the development of different lung cancer-related symptoms. In this review, we summarize recent findings to illustrate the role of P2X receptors in tumor proliferation, progression, metastasis, and lung cancer- related symptoms, providing an outline of potential anti-neoplastic activity of P2X receptor antagonists. Furthermore, compared with opioids, P2X receptor antagonists appear to be innovative therapeutic interventions for managing cancer symptom clusters with fewer side effects.

## Introduction

Lung cancer is one of the most common types of cancer, annually causing about 1.8 million deaths worldwide (1). The early detection of lung cancer and improvements in treatment have increased the survival of patients (2). The number of lung cancer survivors is anticipated to increase to 673,000 by 2026 (3). Cancer survivors experience a complex profile of diverse symptomatology, adversely affecting their quality of life. Consequently, patients complain of a wide variety of symptoms that are mainly categorized as physical and psychological symptoms. Dodd et al. defined the cancer-related symptoms as “symptom clusters” (4). Specifically, in lung cancer patients, respiratory clusters are identified as pain, cough, fatigue, and dyspnea (5, 6). However, cancer patients present multiple symptoms, and lung cancer studies illustrate the complex correlations between different clusters of symptoms. Clinically, opiate-derivative treatment is the mainstay of the drug management of cancer-related symptoms, such as pain, fatigue, cough, and dyspnea (7–9), but benefits relatively few cancer patients. Currently, physicians prescribe opioid therapy without high-quality evidence, which might heighten the potential risk of opioid-induced side effects in patients with cancer, such as sedation, respiratory depression, tolerance development, and gastrointestinal dysmotility (10, 11). Therefore, an in-depth longitudinal exploration of lung cancer related-symptoms is essential to developing a targeted intervention to improve the quality of life of cancer survivors.

Accumulated adenosine triphosphatez (ATP) and other extracellular nucleotides shape tumor microenvironment (TME), significantly influencing cancer proliferation, progression, tumor/immune-cell cross-talk, and related symptoms (12, 13). Accordingly, receptors for extracellular ATP (G-coupled P2Y and P2X ion channels) are involved in driving several functions during tumor initiation and development. To note, the ATP-gated P2X purine family consists of seven subtypes (P2X1–7 receptors) (14), with some being demonstrated to directly or indirectly regulate tumor proliferation, angiogenesis, and dissemination. For example, P2X4, P2X5, and P2X7 receptors exist on the membrane of multiple tumor cells, such as non-small cell lung cancer, colorectal cancer, bladder cancer, renal cancer, as well as haematological malignancies, to promote the proliferation and metastatic potential of the tumor (15–20). In particular, the P2X7 receptor is the subtype associated with cancer proliferation (21), and its activation promotes VEGF-dependent angiogenesis and extracellular matrix degradation *via* protease releasing and cytoskeletal remodeling, playing a prometastatic role in cancer (22–25). A study analyzing P2X7 mRNA expressions in patients with non-small cell lung cancer (NSCLC) revealed an upregulated P2X7 expression in bronchoalveolar lavage fluid of tumor with distant metastases (20). To understand its proliferative and prometastatic roles in tumor, the potential interaction of P2X7R splice variants and cancer cell determination should be discussed. Evidence which appeared on non-pore functional P2X7R (nfP2X7) and P2X7B isoforms in a wide range of tumors suggested that lacking the pore-forming cytotoxic activity enables them to retain a distinct pro-survival trophic property and promote oncologic progression (26, 27). Collectively, the purinergic/adenosinergic system regulates the growth, metastasis, and invasion of cancer, thus rendering P2X purine receptors as potential targets for tumor therapy (13).

More data demonstrated that ATP, its hydrolyzation products, ectonucleotidases, (degrading enzymes, like CD39), and purinergic receptors play a significant role in the modulation of the TME immune component. Extracellular nucleotides and P2 purinergic signaling drive the recruitment of inflammatory cells (such as macrophages, neutrophils, DCs, and microglia) and adjust immunomodulation on tumor sites (12). The purinergic/adenosinergic system modulates cytokine gene expression within the nervous and immune systems and also regulates the secretion of pro-inflammatory cytokines, such as interleukin (IL)-1β, IL-6, and tumor necrosis factor (TNF)-α (28–30). With the collaboration between anti-CD39 and P2X7 activation in the TME, immune cells can bring an antitumor response by P2X7-mediated NLRP3 inflammasome activation and IL-18 release from myeloid cells (31, 32). The relationship of receptor polymorphism and inflammatory responses (including NLRP3 inflammasome activation and IL-1β and IL-8 release) was reported by Hu and colleagues (33). Besides purinergic receptors’ involvement, sensory nerves are also found to be involved in the stimulation of cancer progression, indicating the existence of tumor-nerve interactions. Reportedly, the denervation of vagus nerves and ablation of sensory neurons inhibit tumor initiation and progression in mouse models with cancer (34, 35). Herein, we hypothesize that ATP acts as a pivotal transmitter to convey sensory stimuli from peripheral nerves to the CNS, to activate P2X purine receptors (P2X2, P2X3, P2X4, and P2X7 receptors) expressed on sensory nerve fibers and microglia, to enhance peripheral neural information transmission, as well as to sensitize the CNS (36). A study reinforced this hypothesis that ATP is transported into secretory vesicles in primary afferents and spinal cord by vesicular nucleotide transporter (VNUT) to stimulate related purinergic receptors (i.e. P2X4R), which has been proved in genetic knockout or VNUT inhibitors to relieve neuropathic and inflammatory pain sensation (37). Marked upregulation of P2X4 receptors was detected in C6 glioma tissue; these receptors also activate microglia in the central nervous system (CNS) and tumor-associated macrophages in the peripheral system to mediate inflammatory reactions (38). Taken together, those evidence highlighted the crosstalk between nervous and immune systems *via* P2X pathways. Thus, dissecting the neuro-immune pathways *via* P2X receptors may provide new therapeutic strategies in cancer treatment.

Intriguingly, a high concentration of extracellular ATP in the tumor milieu is able to regulate cancer cell death by exploiting ATP-dependent cytotoxicity (39). Purinergic receptors’ cytotoxic functions are shown under the condition of persistent over-stimulation of high levels of ATP. Prolonged stimulation of P2X7 receptor *via* high dosage ATP leads to the opening of a larger conductance membrane pore, which in turn induces tumor cell death and inhibits tumor growth (15). Sustained opening of the P2X7R macropore stimulated by high extracellular ATP concentration in the TME triggers caspase-3 cleavage and then leads to membrane progression and ultimate cell death *via* different pathways (15, 40). Therefore, it makes sense that the application of P2X receptor agonists, such as ATP and P2X receptor activators, restrains tumor growth. Preclinical cancer models revealed the efficacy of administration of ATP at a high dosage to suppress tumor growth (41, 42). Clinical trials in treating advanced NSCLC patients with intravenous infusion of ATP showed a significant improvement in quality of life and cachexia effects (43). A recent study demonstrated the pro-apoptotic mechanism of P2X7R stimulation, showing that combined with an αPD-1 immune checkpoint inhibitor, HEI3090, as a P2X7R agonist, activates tumor regression in 80% of Lewis lung carcinoma tumor-bearing mice. During tumor inhibition, P2X7R’s cytotoxic activities were described in immune cells, especially dendritic cells, to release pro-inflammatory cytokines (IL-1β and IL-18) *via* an NLRP3-dependent pathway (44). Despite the promising efficacy of P2XR agonist therapy being displayed, side effects should be noticed. In intravenous ATP infusions, dyspnea emerged as the most common side effects, followed by chest tightness, urge to take a deep breath, and cardiac disorders (45). We speculated that ATP agonists activate various P2X receptors, especially P2X3R, P2X4R, and P2X7R, in other tissues or organs to induce a series of symptoms. Their predominant roles are also reported in mediating immune responses and the nervous system (see below). Based on the aforementioned side effects, it is tempting to hypothesize that compared with P2X antagonists/inhibitors, therapies based on P2X agonists would get patients more exposed to more risks of discomforts (i.e. pain sensations, dyspnea, immune system disorders). Whether P2X agonists or antagonists should be adopted depends on multiple factors, including genetic differences (46, 47), different cancer types and onset sites, P2X receptor tumor expression (48), and cancer-associated symptoms (pain, dyspnea, fatigue, etc.). More studies are required to elucidate further use of P2X receptors-targeted therapies in cancer patients in the future.

**Figure 1:**
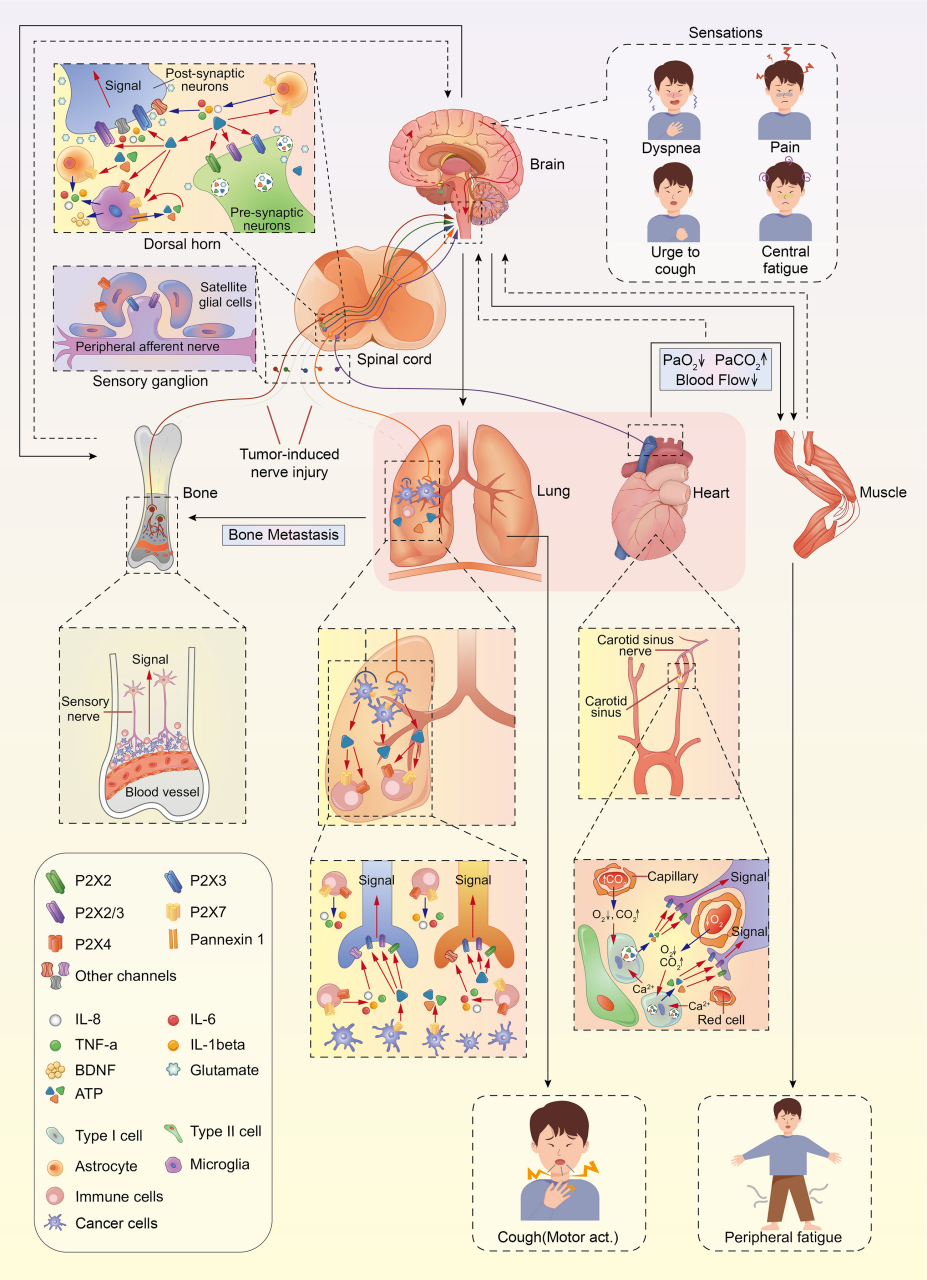
The anatomical and molecular mediators of lung cancer symptom cluster. Ca^2+^, calcium; ATP, adenosine triphosphate; BDNF, brain-derived neurotrophic factor.

A large number of studies have highlighted the role of ATP and P2X purine receptors on the development and progression of cancer. Thus, it could be speculated that a putative P2X-dependent mechanism affects cancer-related symptoms, including pain, fatigue, cough, and dyspnea. Herein, we summarize the related findings on the potential correlation between different types of cancer symptom clusters and P2X purine signaling, providing an outline for the potential treatment of distressing cancer-related symptoms using P2X receptor antagonists (**Figure 1**).

## Cancer-Related Pain

Half of the cancer patients may experience different levels of pain, especially those with the middle and advanced stages of cancer (49). The growth, proliferation, migration, and invasion of tumors compress and damage peripheral nerves, and then sensitize peripheral sensors and transmit the sensation of pain (50). Bone cancer pain may be an applicable example, which results from primary bone cancer and the metastasis of other common cancers, such as lung cancer, breast cancer, and prostate cancer. Tumor infiltration in the bone compresses and damages sensory nerve fibers, which in turn aggravates the pain. Furthermore, immune cells (such as lymphocytes and macrophages) play a critical role in the tumorigenic site by releasing inflammatory cytokines (such as TNF-a, IL-1, IL-6, and substance P (SP) (51). Based on these perspectives, cancer pain is related to nociceptive pain, neuropathic pain, and inflammatory pain.

In the tumor microenvironment, ATP and P2X receptors were detected in patients with cancer pain (52). Increased extracellular ATP triggers pain *via* activation of different P2X receptors involved in nociceptive, neuropathic, and inflammatory pain. Specifically, ATP-gated P2X3 and P2X2/3 receptors selectively expressed in primary afferent nerves (vagal C fibers and Aδ-fibers) and afferent sensory neurons (i.e. dorsal root ganglia, trigeminal nerve, inferior ganglion of the vagus nerve, and glossopharyngeal nerve) (53, 54) participate in the development and transmission of pain signals with respect to chronic inflammatory pain, neuropathic pain, and cancer pain (55, 56). Several studies have shown upregulation of P2X3 receptors in the dorsal root ganglion of animals that experienced bone cancer pain (57–59). Data also support that homomeric P2X3 and heteromeric P2X2/3 receptors are involved in the generation of bone cancer pain perception (60, 61). A-317491, a P2X3 receptor antagonist, could efficiently relieve cancer-induced bone pain related to early-stage tumors. However, it had limited effects on inhibiting the progression of the tumor from an early to an advanced stage (62). Similarly, suramin, a broad spectrum P2 receptor antagonist, reduced prostate-specific antigen levels, pain, and disease progression but the mechanism of pain reduction remains unknown. Another selective P2X3 and P2X2/3 receptor antagonist, AF-353, had an efficacious pain-easing function but could not prevent cancer-induced bone destruction. With the application of *in vivo* electrophysiology, AF-353 administration on the spinal cord of animals with bone cancer reduced the neuronal hyperexcitability to mechanical and chemical stimuli, suggesting that the central nervous system was involved in the cancer pain (59).

Furthermore, P2X4 expressed on microglial cells within the CNS and P2X7 expressed on nerve cells as well as macrophages in the periphery participate in the etiology of cancer pain. The upregulation of P2X4 receptors in spinal microglia induces the release of brain-derived neurotrophic factor (BDNF), a short-term promoter of neuropathic pain (63). P2X7 receptors expressed in spinal microglia participated in the development of advanced cancer pain by modulating ATP release and pro-inflammatory cytokine production (64, 65). C57BL/6J P2X7 receptor knockout (KO) mice had absent responsiveness to thermal and mechanical stimuli, and their macrophages did not secrete cytokines (for example, IL-1β) under the stimulation of lipopolysaccharide (LPS), indicating that P2X7 receptors enhance nociceptive transmission by elevating the secretion of cytokines *via* macrophages (66). Conversely, BALB/cJ P2X7R KO mice had pain-related behaviors after bone cancer induction. Compared with wild-type counterparts, they presented an earlier onset for pain-related behaviors and a significantly severe pain phenotype (67). Different genetic backgrounds of mice strains might account for discrepant results. C57BL/6 and BalbcJ P2X7R deficient mice might express different P2X7 polymorphisms that would cause variability in phenotypes and presentations (e.g. pain-related behaviours, bone remodeling) in comparison of WT type littermates (47, 68–71). Besides animal models, two human chronic pain cohort (one with pain after mastectomy and the other with osteoarthritis), revealed a genetic association between intensive chronic pain and the hyperfunctional variant of His 270 (rs7958311) allele of P2X7R, suggesting that selective blockade aiming at P2X7R pore formation also seems to be an innovative and personal therapeutic strategy for individual chronic pain (47).

The knockdown of P2X7 receptors with the utilization of selective or specific antagonists (A-438079, A-740003, and A-839977) benefits cancer pain by reducing the release of cytokine, such as Il-1β (66, 72, 73). Another study reported that acute treatment with A-438079 failed to reduce pain-related behaviors in a mouse model of bone cancer pain (67). Current evidence suggests that the P2X4 receptor expressed on microglia induces spinal inflammatory pain and neuropathic pain and releases cytokines (74, 75). However, only a few studies have reported the role of P2X4 receptors in the treatment of cancer pain. A recent study has shown that RS-504393, a selective antagonist of the chemokines C-C motif receptor 2 (CCR2), reduced the expression of P2X4R in the spinal cord and relieved bone cancer pain, rendering P2X4R a potential target for cancer pain therapy (76).

Kyushu University and Nippon Chemiphar Co. Ltd initiated a Phase 1 clinical trial of NC-2600 in 2016 to elucidate its efficacy in chronic neuropathic pain (to date, there is no information on the structure or preclinical data). Although P2X7 antagonists, such as AZD9056 and CE-224,535 were well tolerated in Phase 1 clinical studies, they showed insignificant efficacy in treating rheumatoid arthritis in Phase 2 clinical trials. Another P2X7 antagonist, AZD9056, has the therapeutic potential to decrease Crohn’s disease activity index along with improvement in chronic abdominal pain, as shown in a Phase 2a clinical study of Crohn’s disease (**Table 1**). However, only a few randomized, double-blind, placebo-controlled clinical studies have demonstrated the efficacy and safety of P2X receptors in treating cancer pain. Collectively, the usage of P2X antagonists might present a promising therapeutic strategy for cancer-associated pain compared to the current, commonly-used agents, such as opioids (53).

**Table 1:** Clinical Studies of P2X Receptor Antagonists.

Targets	Compound	Trial Identifier	Responsible Party	Development	Indications	Estimated Completion
P2X4R	NC-2600	Pharmaceuticals and Medical Devices Agency (PMDA)	Kyushu University, Nippon Chemiphar Co., Ltd	Phase 1	Neuropathic pain	Completed (no results posted), www.chemiphar.co.jp
P2X3R	AF-219/MK-7264/Gefapixant	NCT03449134 (COUGH-1)	Merck Sharp and Dohme Corp.	Phase 3	Chronic cough	Completed (published in an abstract form)
		NCT03449147 (COUGH-2)	Merck Sharp and Dohme Corp.	Phase 3	Chronic cough	Completed (published in an abstract form)
		NCT02477709	Afferent Pharmaceuticals, Inc.	Phase 2	Idiopathic Pulmonary Fibrosis (IPF)	Completed (results posted online)
		NCT02502097	Afferent Pharmaceuticals, Inc.	Phase 2	IPF-associated cough	Completed (results posted online)
	BAY 1817080	NCT04562155	Bayer	Phase 1/2	Chronic cough	Primary Completion: Jun, 2021 Study Completion: Jul, 2021
		NCT03310645	Bayer	Phase 1/2	Chronic cough	Completed (no results posted)
		NCT04614246	Bayer	Phase 2	Pain related to endometriosis	Recruiting Primary Completion: Aug. 2022 Study Completion: Sep. 2023
		NCT04641273	Bayer	Phase 2	Diabetic neuropathic pain	Recruiting Primary Completion: Jun.2023 Study Completion: Jul.2023
	BLU-5937	NCT03979638 (RELIEF)	Bellus Health Inc.	Phase 2	Chronic cough	Terminated early because of the impact of COVID-19 on the trial activities (no results posted)
	BAY1902607	NCT03535168	Bayer	Phase 1/2	Chronic cough	Completed (no results posted)
	S-600918	NCT04110054	Shionogi Inc.	Phase 2b	Chronic cough	Completed (no results posted)
P2X7R	AZD9056	Eudra-CT Number: 2005- 002319-26, Sponsor Protocol Number: D8830C00002	AstraZeneca AB	Phase 2a	Chronic abdominal pain related to Crohn’s disease (CD)	Completed (results posted online)
		NCT00520572	AstraZeneca AB	Phase 2	Rheumatoid Arthritis	Completed (results posted online)
	CE-224,535	NCT00628095	Pfizer Inc.	Phase 2/3	Rheumatoid Arthritis	Completed (no results posted)
		NCT00418782	Pfizer Inc.	Phase 2	Osteoarthritis pain	Completed (no results posted)
	GSK1482160	NCT00849134	GlaxoSmithKline	Phase 1	Inflammatory pain	Completed (no results posted)
	JNJ-54175446	NCT04116606	CCTU-Core, Cambridgeshire and Peterborough NHS Founation Trust	Phase 2	Major depression disorder, inflammation	Recruiting
						Primary Completion: May, 2021
						Study Completion: Dec. 2021
		NCT02902601	Janssen Research and Development, LLC.	Phase 1	Major depression disorder	Completed (no results posted)

## Cancer-Related Cough

A persistent cough is a common symptom affecting over 50% of patients with lung cancer with a considerably adverse impact on quality of life (77). Cough can be evoked by activation of vagal afferent nerves innervating the airways and lungs—C-fibers (chemically) and Aδ-fibers (mechanically) (78, 79). In lung cancer, coughing is often provoked by the tumor (especially endobronchial neoplasms)-stimulating bronchial receptors and/or by the inflammatory responses of the tumor activating afferent nerve fibers in the airways (80). In one study, lung cancer related-cough remained refractory while other cancer symptoms were managed adequately in clinical practice (81). Moreover, chronic dry cough is present in about 25–50% of patients after undergoing lung cancer surgery (82, 83). Despite such high prevalence, current practice on treating cancer-related cough is empirical without high-quality evidence. Oral opioids are widely used in managing patients with cancer but with significant side effects (81).

ATP-gated P2X3 receptors are localized on the terminals of vagal C fibers innervating the lungs and airways and are activated by ATP released into the airway in guinea pigs (84, 85). In addition, the terminals of Aδ-fibers express P2X3 receptors (53). In the airways and lungs, ATP acts as a trigger of the cough reflex *via* stimulation of P2X3 and P2X2/3 receptors expressed on the vagal sensory neurons central to the cough reflex (86, 87). Kamei et al. observed that when exposed to tussive stimuli, such as ATP and histamine aerosols, cough reflex sensitivity was enhanced *via* P2X receptor signals in guinea pigs. This effect could be attenuated by pretreatment with TNP-ATP, a potent P2X3 antagonist, in guinea pigs (88, 89). Morice et al. reported the ability of the P2X3 receptor antagonist gefapixant (formerly named AF-219 and MK-7264) in alleviating cough induced by ATP and ultrasonically-nebulized distilled-water, but not capsaicin and citric acid (90). Bonvini et al. identified that TRPV4-ATP-P2X3 interaction was involved in cough hypersensitivity in guinea pig conscious cough models (91). These findings suggest that the P2X3 pathway might underlie cough hypersensitivity in chronic refractory cough, supporting a potential therapeutic role in the treatment of lung cancer-induced chronic cough.

In the tumor microenvironment, elevated levels of ATP and adenosine due to degradation of extracellular ATP were observed during cancer development, which might subsequently activate purinergic receptors that are fed to the central nervous system (12). Recent studies have demonstrated the efficacy of P2X receptor antagonists in the management of chronic cough and showed an association between ATP and cough *via* purinergic signaling. Gefapixant, a first-in-class selective antagonist of the P2X3 and P2X2/3 receptors, significantly inhibited ATP-evoked cough in patients with chronic cough (90). Furthermore, gefapixant showed a significant reduction in cough frequency, as assessed by randomized, double-blind, placebo-controlled Phase 2 trials (92, 93). Recently, two Phase 3 randomized controlled trials (COUGH-1 and COUGH-2) of gefapixant in refractory chronic cough and unexplained chronic cough, evaluating more than 2000 patients, have been completed (94). Compared to placebo, treatment with 45 mg gefapixant demonstrated a significant reduction in 24-h cough frequency in patients (95). However, the loss of taste was an adverse event accompanied by incremental doses of gefapixant (92, 93). Reportedly, the adverse events with gefapixant 45 mg were mostly related to altered taste (95).

Another P2X3-receptor antagonist, BLU-5937 demonstrated safety and tolerability in the Phase 1 clinical trial (96), while the Phase 2 clinical trial was delayed due to the COVID 19 pandemic. According to the current data released by the sponsor, this trial failed to meet the primary endpoints for any doses, although BLU-5937 was well-tolerated and its efficacy in reducing awake cough frequency was also found in a subgroup of patients with high cough counts at baseline. Two other P2X3-selective antagonists, BAY 1817080 and S-600918, have shown antitussive effects in completed Phase 2 trials (97, 98). BAY1902607 underwent a Phase I/II proof-of-concept clinical trial. Although it has been announced as complete, the results have not yet been posted (**Table 1**).

In light of published and ongoing promising data with at least four clinical programs involving P2X3 receptor-antagonists in treating chronic refractory cough (90–98), and with extracellular ATP and its receptors having been shown to modulate tumor development, invasion progression, and tumor microenvironment, P2X receptor antagonists may be expected also to exert an antitussive effect in lung cancer-related cough. Further studies in this area are eagerly awaited.

## Cancer-Related Fatigue

Cancer-related fatigue (CRF) is one of the cancer cluster symptoms that affects up to 90% of patients with lung cancer (99). This distressing, persistent, and subjective sense of tiredness or exhaustion might be a manifestation of cancer or the side effects of its treatment. Central and peripheral mechanisms could be involved in the development of CRF. The central hypotheses consist of pro-inflammatory cytokine signaling dysregulation, hypothalamic-pituitary-adrenal (HPA) axis disruption, circadian rhythm disorder, serotonin dysregulation, and vagal afferent activation, while, peripheral mechanisms may include ATP dysregulation, muscle metabolism, and effects on contractile properties (100).

Accumulating evidence suggests that inflammation is a common link between CRF and cancer pain. Bone cancer pain enhances the levels of inflammatory factors (TNF-α, IL- 1β, and IL-6) *via* activation of glial cells and central sensitization in the spinal cord (101). Similarly, compared to non-fatigued survivors or healthy controls, fatigued cancer patients had significantly higher levels of pro-inflammatory cytokines, such as C-reactive protein (CRP), IL-1β, IL-6, and TNF-α (102, 103) in the tumor microenvironment (104, 105) or from anti-tumor treatment (radiation therapy or chemotherapy) (106, 107). Enhanced cytokine activity in the periphery might be conveyed to the brain, which in turn would induce central fatigue by altering the serotonin pathway in the brain, influencing the HPA axis, dysregulating circadian rhythms, and inducing vagal afferent activation (108, 109). Symptoms such as fatigue, depression, and loss of appetite, may be the result of peripheral inflammation, mediated by pro-inflammatory cytokines (110). These cytokines also underlie the development of anemia (111), cachexia (112), and depression (113), which may contribute to CRF. Pain and fatigue share similar inflammatory mechanisms. Thus, fatigue management might utilize P2X antagonists, such as P2X7 selective or specific antagonists which have been demonstrated to ameliorate pain and inhibit cytokine release (66, 72, 73).

In terms of a peripheral hypothesis, limited evidence is available on cancer-related fatigue. However, most patients with cancer suffer from weight loss and/or loss of appetite, which might alter muscle protein metabolism. Peripheral fatigue might be manifested by the inability of muscle, with altered muscle protein metabolism, ATP dysregulation, and contractile properties, to respond to central stimulation (114, 115). The combined products of muscle contraction (ATP, proton, and lactate) activate the sensory neurons innervating skeletal muscle, which might project to the central nervous system *via* sensory neurons and evoke a sensation of fatigue. Moreover, in an animal model, ATP released from fatigued muscle activates muscle macrophages, which subsequently release IL-1β to produce hyperalgesia. The blockade of P2X4 receptors in muscle inhibits the development of such hyperalgesia (116).

Pilot data suggest that P2X receptors may act as valid pharmacological targets. To date, the correlation between P2X receptors and cancer-related fatigue has been minimally investigated, and hence, further study of the efficacy of P2X antagonists in treating cancer-related fatigue is needed.

## Cancer-Related Dyspnea

Moderate or severe dyspnea is reported in 20–80% of cancer patients. Dyspnea is a common symptom in patients with advanced cancer, accompanied by cough due to primary lung cancer in the airway or metastasis of other cancers to the bronchus. Especially, intraluminal tumor in the trachea or a mainstem bronchus activates cough receptors and mechanically obstructs airflow to elicit the sensation of dyspnea (117). Dyspnea is a multifactorial sensation associated with central, peripheral, and chemoreceptor modulation (118). Notably, vagal nerves innervating both the airways and lungs and transmitting to the sensorimotor cortex are capable of inducing dyspnea (119). Several studies have indicated that vagal C-fibers are of dyspneic origin and can be triggered by ATP and adenosine. Furthermore, some Aδ stretch fibers can be directly stimulated by ATP similar to C-fibers (53, 120, 121). In clinical trials, dyspnea could be evoked by the direct C-fiber activators ATP and adenosine during bronchial challenge testing (122). Inhaling ATP and adenosine induced dyspnea in subjects with asthma (123) and COPD (124). Additionally, intravenous injection of adenosine induced distressing symptoms such as dyspnea, hyperventilation, urge for deep breathing, and chest tightness in healthy volunteers (125), and pre-terminal cancer patients (45, 126). Taken together, extracellular ATP is likely to give rise to dyspnea, and thus may be a potential target for the treatment of dyspnea.

Hypoxemia may be one of the factors stimulating ventilation and producing dyspnea in lung cancer patients (127). Furthermore, central cardiovascular and respiratory neuronal networks are intertwined during and post-hypoxia (128). In knockout P2X2 mice, the activation of the chemoreceptor of the carotid sinus nerve transmits information about arterial pO_2_ to the respiratory centers of the brain to mediate ventilatory responses to hypoxia. The P2X2 receptor antagonist PPADS was shown to block neural discharge evoked by hypoxia (129). Furthermore, Zhang et al. reported that suramin, a P2X receptor antagonist, combined with a nicotinic Ach receptor antagonist, blocked the hypoxia-induced increase in chemoreceptor afferent neuronal discharge (130).

Collectively, these studies suggest that the inhibition of specific ATP signal transduction pathways, such as with P2X3 receptor antagonists in the lungs, constitutes an attractive target for the development of new therapies to ameliorate dyspnea. However, additional randomized, double-blinded, placebo-controlled clinical studies are required to confirm whether P2X antagonists are able to eliminate or improve cancer-induced dyspnea.

## Conclusions

Cancer-related symptoms may significantly and adversely affect patients with lung cancer, causing a marked diminution in the quality of life. Hence, the potential therapeutic roles of P2X receptors in cancer as well as cancer-related symptoms need to be further elucidated. Intratumoral ATP, ATP-P2X receptors, and the vagus nerve are involved in the neuro-immune interactions in cancer. Increased extracellular ATP and its associated compounds activate P2X receptors expressed on bronchopulmonary nerves to evoke unpleasant sensations (such as the urge to cough, dyspnea, chest tightness, fatigue, and pain) and stimulate/modulate reflexes (such as cough, bronchoconstriction, respiratory rate, and inspiratory drive). Various cancer-related symptoms may share similar pathogenic mechanisms. To date, a significant clinical investigation has established a role for P2X receptor antagonists in the treatment of chronic cough and cancer-related pain. Thus, a basis has been formed for the further evaluation of potential therapeutic roles for P2X antagonists in the treatment of other cancer-induced symptoms.

## Author Contributions

YM, ZG, and WY drafted the manuscript. NZ, PD, and RC designed and revised the manuscript. All authors contributed to the article and approved the submitted version.

## Funding

This study was supported by grants from the National Natural Science Foundation of China (81870079) and National Outstanding Youth Incubation Program of Guangzhou Medical University(2020-207).

## Conflict of Interest

The authors declare that the research was conducted in the absence of any commercial or financial relationships that could be construed as a potential conflict of interest.

## Abbreviation

ATP, adenosine triphosphate; BDNF, brain-derived neurotrophic factor; Ca^2+^, calcium; CNS, central nervous system; CRF, Cancer-related fatigue; CRP, C-reactive protein; HPA, hypothalamic-pituitary-adrenal; NSCLC, non-small cell lung cancer; IL, interleukin; KO, knockout; LPS, lipopolysaccharide; SP, substance P; TNF, tumor necrosis factor; TME, tumor microenvironment; VNUT, vesicular nucleotide transporter.
